# Coil Embolization for a New Ulcer-Like Projection Near the Distal Anastomosis After a Hemiaortic Arch Replacement

**DOI:** 10.7759/cureus.71559

**Published:** 2024-10-15

**Authors:** Eiji Nakamura, Kazuyoshi Takagi, Hiroyuki Otsuka, Shinichi Hiromatsu, Eiki Tayama

**Affiliations:** 1 Division of Cardiovascular Surgery, Department of Surgery, Kurume University School of Medicine, Kurume, JPN; 2 Division of Cardiovascular Surgery, Department of Surgery, Kurume University School of medicine, Kurume, JPN

**Keywords:** aortic dissection, coil embolization, endovascular therapy (evt), minimally invasive therapy, ulcer-like projection

## Abstract

Ulcer-like projections (ULPs) with a tendency to enlarge are at risk of aortic events such as new dissection, aneurysmal formation, or rupture and require therapeutic intervention. However, what should be done after open chest surgery when standard thoracic endovascular aortic repair (TEVAR) cannot be performed is debatable. Here, we present a case of coil embolization of a newly enlarged ULP that was not amenable to TEVAR following a hemiarch aortic arch repair. A 68-year-old male with a history of ascending and hemiaortic arch repair for acute type A aortic dissection presented with a chief complaint of chest pain three months prior to presentation. A post-type A dissection ULP remained in the aortic arch but had enlarged over the three months. Therapeutic intervention was planned to avoid aortic events; however, standard and fenestrated TEVAR were going to be anatomically challenging. Since the patient was in poor general condition after diverticulitis and stroke, reopened total arch replacement and total debranching TEVAR were avoided. The neck of the ULP was narrow and had a small volume; therefore, we assessed that the coil-packing method could embolize the ULP. Coil embolization was successful, and the patient had no postoperative complications. A computed tomography scan at the remote stage showed no recanalization or enlargement, and the patient was stable. Coil embolization may be attempted for arch ULP that can enlarge if the ULP can be embolized with intra-aneurysmal packing when it is difficult to perform a reopen surgery or TEVAR, including standard zone II, total debranching, and fenestrated TEVAR.

## Introduction

Ulcer-like projections (ULPs), which have a propensity for enlargement, can precipitate aortic events [1,2]. Miyahara et al. defined ULPs in acute type B aortic dissection as any focal blood-filled pouch projecting into a thrombosed false lumen [3]. In their study, patients with ULPs were more prone to a poor prognosis due to late aortic events. The incidence of ULPs in the residual arch after aortic dissection surgery remains uncertain; however, their natural history suggests potential late complications. In cases of uncomplicated acute type B aortic dissection, preemptive thoracic endovascular aortic repair (TEVAR) to close the entry site is increasingly being recognized as effective for averting remote aortic events [4,5]. Although this approach may also hold promise for postoperative ULPs and anastomotic pseudoaneurysms, its indication depends on the anatomical conditions, especially in the aortic arch. Coil embolization may be considered an alternative treatment option. Some reports suggest that minimally invasive endovascular treatment using coil embolization can result in decreased mortality and morbidity in patients with ascending aortic pseudoaneurysms after open aortic repair [6,7]. However, there are no documented cases of coil embolization for postoperative ULPs in the aortic arch. Here, we present a case of coil embolization of a newly enlarged ULP that was not amenable to TEVAR following a hemiarch aortic arch repair.

## Case presentation

**Figure 1:**
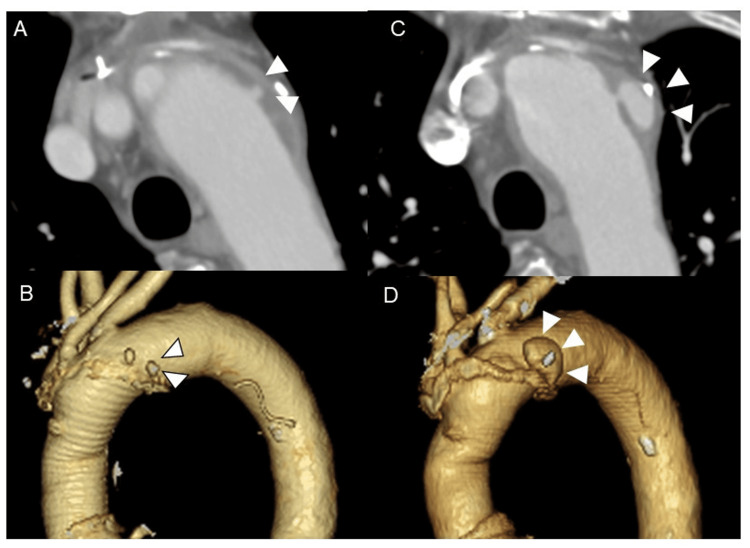
The CT findings immediately after the hemiarch replacement and three months later The CT scans immediately (A, B) and three months postoperatively (C, D) show an enlarged ULP (white arrowheads) from 5 × 5.5 mm to 17 × 9 mm near the distal anastomosis after hemi-aortic arch replacement. ULP: ulcer-like projection; CT: computed tomography

A 68-year-old male with a history of ascending and hemiaortic arch repair for acute type A aortic dissection presented with a chief complaint of chest pain three months prior to presentation. During the perioperative period of aortic surgery, the patient experienced a stroke, presenting with left upper extremity paraparesis and left hemispheric neglect. The postoperative CT revealed a new ULP measuring 5 × 5.5 mm at the lesser curvature of the aortic arch (Figures 1A, 1B). The patient continued to receive strict antihypertensive medication. Precisely three months after the aortic surgery, the patient presented to our hospital with right lower abdominal pain and was diagnosed with diverticulitis of the colon. The CT scan obtained at this time showed enlargement of a previously known ULP measuring 17 × 9 mm, prompting the decision to reintervene because of ULP expansion within a short period and the risk of rupture (Figures 1C, 1D).

Additional interventions were planned following the treatment for colonic diverticulitis. There was no recent history of fever, chest pain, back pain, or any other symptoms such as hoarseness. Standard zone 2 TEVAR was anatomically challenging because of the position of the proximal end of the ULP and the left common carotid artery. This made it impossible to secure a sealing zone of > 20 mm, making the process contraindicated. Fenestrated TEVAR with the Najuta stent graft system (SB-Kawasumi Laboratories, Inc., Kanagawa, Japan) was also anatomically challenging because of the use of inner and outer felt strips for distal anastomotic stump plasty during type A dissection surgery, which could lead to endoleak from the fenestration.

**Figure 2:**
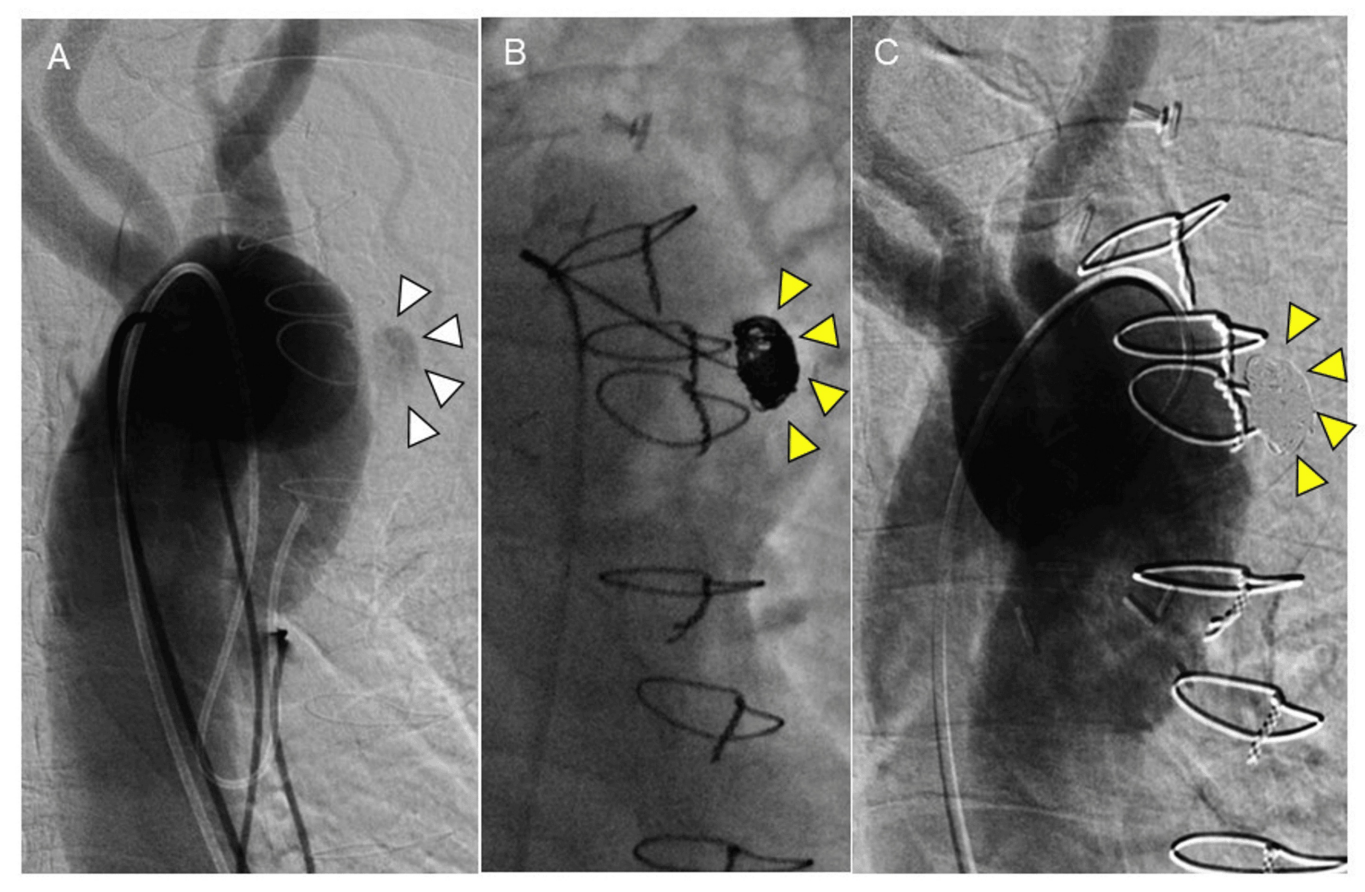
Angiographic findings during coil embolization of the ULP Angiography showing the ULP (white arrowhead) at the lesser curvature of the aortic arch (A) and the ULP with loss of contrast by coil embolization (yellow arrowheads) (B, C) ULP: ulcer-like projection

The patient was in poor general condition due to the sequelae of stroke and colonic diverticulitis. Since the reopened chest and zone 0 TEVAR with total debranching were considered excessively invasive, coil embolization for the ULP was scheduled. Coil embolization of the ULP was performed under local anesthesia using a right common femoral artery (CFA) approach. A 5Fr sheath was inserted, and a 4Fr catheter (Hirai, Medikit Co. Ltd., Tokyo, Japan) was positioned at the neck of the ULP. Subsequently, a microcatheter (2.0/2.8-Fr, Light House, Piolax Medical Devices, Inc., Kanagawa, Japan) was coaxially inserted into the ULP using a 0.016-inch guidewire (Chaser, Piolax Medical Devices, Inc.). The ULP was then filled with Target XXL 360 (Stryker Neurovascular, Fremont, CA, USA; 14 mm×50 cm, 10 mm×50 cm), followed by embolization using Target XL 360 soft coils (Stryker Neurovascular, Fremont; 5×10×2 and 4×12 cm) as finishing coils (Figures 2A, 2B). The final contrast imaging revealed a loss of contrast within the ULP (Figure 2C).

**Figure 3:**
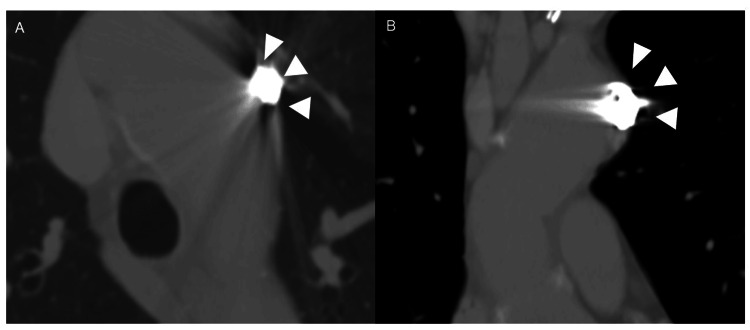
The CT findings three years after coil embolization of the ULP The CT findings three years after coil embolization of the ULP (A, B) show that the ULP had almost disappeared, with no residual blood flow (white arrowheads). ULP: ulcer-like projection; CT: computed tomography

The patient was discharged without any postoperative complications after a short period. A CT scan performed on an outpatient basis three years postoperatively revealed that the ULP had disappeared, with no obvious tendency for enlargement or residual blood flow (Figures 3A, 3B).

## Discussion

Patients with ULPs after acute type B dissection are more likely to have poor prognosis because of late aortic events [3]. The incidence of ULPs in the residual arch after aortic dissection surgery remains unknown; however, the natural course of ULPs suggests that they may contribute to late complications. Rapid enlargement of ULPs near the distal anastomosis within a relatively short period is considered a high-risk factor for developing aortic events; thus, surgical intervention was warranted for this patient.

Treatment for ULPs includes reopening surgery, TEVAR, and endovascular therapy such as coil embolization. Although reopening surgery is still considered the gold standard, it has low activity on the day after stroke and a high mortality rate [8]. In this case, reopening surgery was considered a high-risk procedure because of the patient’s low activity and frailty. Preemptive TEVAR for entry closure is increasingly accepted to protect against remote aortic events in patients with uncomplicated acute type B aortic dissection [4,5]. Similarly, this approach may benefit patients with new ULPs that tend to enlarge after a hemiaortic arch replacement for acute type A aortic dissection. In this case, standard zone 2 TEVAR was difficult because of the lack of a proximal sealing zone at the lesser curvature of the arch. Fenestrated TEVAR with the Najuta system (SB-Kawasumi Laboratories, Inc.) was also going to be difficult, expected to lose the sealing zone due to the step created at the distal anastomotic stump plasty with inner and outer felt strips, and induce an endoleak from the gap. The gold standard therapy for postoperative ULPs is reopening surgery, or TEVAR. If anatomical indication exists, even postoperatively, TEVAR is the first choice due to its minimally invasive nature. When TEVAR is not feasible due to anatomical conditions, the alternative is reopening surgery, but it takes longer, causes more blood loss, and has a higher mortality and complication rate. We investigated the possibility of endovascular embolization as an alternative.

The coil embolization procedure is straightforward, can be performed under local anesthesia, does not require specialized equipment, and can be completed quickly with minimal hospitalization, thereby enabling minimally invasive procedures. Coil embolization has been documented as a viable treatment option for anastomotic pseudoaneurysms following open aortic repair for acute aortic dissection [6, 7], traumatic ULPs in the abdominal aorta, and pseudoaneurysms after stenting of aortic arch coarctation in patients with a history of median sternotomy [9, 10]. Stent graft implantation for penetrating atherosclerotic ulcers (PAU) of the abdominal aorta combined with coil embolization of the PAU to prevent type II endoleaks has also been reported [11]. Although the safety and efficacy of coil embolization for aortic diseases have been established, several challenges must be addressed before applying this technique to ULPs on the aortic arch after hemiarch replacement in clinical practice. Unfortunately, there are no reports showing the long-term efficacy or safety of coil embolization for postoperative ULPs. Therefore, long-term follow-up is essential, and the indications must be strict.

Complications specific to coil embolization (e.g., distal embolization and unexpected branch occlusion due to coil migration and infection) require careful consideration [12]. The anatomical morphology of ULPs is crucial for preventing these complications. Ulcer-like projections with narrow necks and small mouths, as demonstrated in our case, result in reduced coil migration and minimize the need to place multiple coils for occlusion. Ulcer-like projections with wide necks and large mouths would increase the risk of coil migration and unexpected branch embolization, and the packing itself would be more likely to be inadequate due to its large volume, and the number of coils required for packing would also increase. Therefore, we advocate early coil embolization because delayed therapeutic intervention may lead to enlargement of the neck and mouth, thereby increasing the risk of coil embolization. Technical considerations are also important in this regard. The coaxial catheter system effectively supported the ULP neck, allowing safe catheter placement without concerns regarding coil migration during embolization [13]. Additionally, the target coil, which is detachable and capable of repositioning until final deployment, maintained high shape stability despite its soft shape, with no observed kickback. Although long-term durability remains challenging owing to limited data, this case demonstrates safety over at least a three-year period.

## Conclusions

Ulcer-like projections with rapid enlargement require therapeutic intervention to avoid aortic events. Coil embolization may be considered an alternative treatment option. In the previous literature, there have been no documented cases of coil embolization for postoperative ULPs in the aortic arch, but there have been reports of its use for pseudoaneurysms, traumatic ULPs, and PAUs with good results. Our case demonstrates that coil embolization for ULPs after a hemiarch aortic replacement, particularly in patients at risk of aortic events, is beneficial for those unable to undergo reoperation or TEVAR because of their overall health status or anatomical constraint. However, further studies are needed to prove the long-term therapeutic efficacy and safety of this technique.
